# Routing cancer immunology and immunotherapy from the lab to the clinic 4–5 th March 2014, Center for Applied Medical Research and University Clinic, Pamplona, Spain

**DOI:** 10.1186/1479-5876-12-202

**Published:** 2014-07-24

**Authors:** M Ángela Aznar, Ignacio Melero, José I Quetglas

**Affiliations:** 1 Center for Applied Medical Research, CIMA and University Clinic, University of Navarra, Pamplona, Spain

## Abstract

New approaches to generate effective anticancer responses by either inducing immune responses or inhibiting immunosuppression are under development to improve efficacy in patients. On March 4-5^th^, 2014, a symposium was held in Pamplona, Spain, to report the new strategies showing preclinical and clinical results regarding translational research efforts on the topic. Participants interacted through oral presentations of 15 speakers and further discussions on topics that included novel therapeutic agents for cancer immunotherapy, viral vectors and interferon-based approaches, experimental tumor imaging and immunostimulatory monoclonal antibodies. Promising agents to target cancer cells and therapeutic approaches that are under translation from bench to patients were presented.

## Report

The many and excellent results of cancer immunotherapy strategies are dazzling in the eyes of clinical oncologists. Immunomodulation and adoptive T cell therapies are taking the limelight from years of obscure and busy basic, translational and clinical research. The meeting that we report was aimed to be most translational, mixing together presentations on new agents, mechanisms of action, clinical results and new problems from bench to bed (Table 1). We need researchers to team in asking and answering the questions which will let us make the most of the ongoing immunotherapy revolution. As Walt Disney said once: “Let’s never forget that everything started with a mouse”. Indeed, most of the talks in this meeting started with mouse data and many of them finished with human results. This significant trend is telling us that translation is paying off.

**Table 1:** List of the speakers, affiliations, titles of the talks and their corresponding section in the report

**Speaker**	**Affiliation**	**Talk title**	**Section**
**Guido Kroemer**	Institut Gustave Roussy, France.	Interferon, viral mimicry and chemotherapy. The hallmarks of immunogenicity.	Viral vectors and interferon-based approaches in cancer immunotherapy.
**Cristian Smerdou**	Center for Applied Medical Research (CIMA), Spain.	Alphavirus vectors for cancer immunotherapy.	
**Sandra Hervás-Stubbs**	Center for Applied Medical Research (CIMA), Spain.	New roles for the Type I IFN system in cancer immunology.	
**David Escors**	Navarrabiomed, Spain.	*Ex vivo* differentiation of cancer-specific myeloid-derived suppressor cells and their use in cancer immunotherapy.	
**David Sancho**	Centro Nacional de Investigaciones Cardiovasculares Carlos III (CNIC), Spain.	A dendritic cell receptor for damaged self is crucial for anti-viral immunity.	
**Bettina Weigelin**	Radboud University Nijmegen, The Netherlands.	Dynamic imaging reveals serial killing of cancer cells by CTL: New strategies to overcome tumor resistance niches.	Experimental tumor imaging in immunotherapy.
**Philippe Bousso**	Institut Pasteur, France.	*In vivo* imaging of immune tumor rejections.	
**Ignacio Melero**	Center for Applied Medical Research (CIMA), Spain.	Making the most of CD137 and IL-15 for cancer immunotherapy.	Immunostimulatory monoclonal antibodies.
**Bruno Paiva**	Center for Applied Medical Research (CIMA), Spain.	Monitoring myeloma in minimal residual disease: a window of opportunity for immunotherapy.	
**Paolo Ascierto**	National Tumor Institute Fondazione G. Pascale, Italy.	Biomarkers for check point blockade.	
**Kohrt Holbrook**	Stanford University, USA.	Stimulation of Natural Killer Cells with an Anti-CD137 Antibody Enhances the Efficacy of Trastuzumab, Cetuximab & Rituximab.	
**Noelia Casares**	Center for Applied Medical Research (CIMA), Spain.	Targeting FOXP3. Development of inhibitors for immune modulation.	Novel therapeutic agents for cancer immunotherapy.
**Marisol Soengas**	Spanish National Cancer Research Centre (CNIO), Spain.	New animal models for gene discovery and drug screening.	
**Fernando Pastor**	Center for Applied Medical Research (CIMA), Spain.	CD137 and CD28 agonist aptamers.	
**Luis Álvarez-Vallina**	Hospital Universitario Puerta de Hierro, Spain.	New antibody platforms in immunotherapy and anti-angiogenesis.	

### Viral vectors and interferon-based approaches in cancer immunotherapy

In the opening keynote lecture of the symposium, Guido Kroemer (Institut Gustave Roussy, France) described the immune-dependent effects of chemotherapy. Physiological cell death, which occurs as a continuous byproduct of cellular turnover, is non-immunogenic or even tolerogenic, thereby avoiding autoimmunity. However, cancer cell death elicited by radiotherapy and some chemotherapeutic agents such as anthracyclines and oxaliplatin can be immunogenic. Immunogenic cell death is characterized by the early cell surface exposure of calreticulin, which determines the uptake of tumor antigens by dendritic cells (DC). The late release of the protein high mobility group box 1 (HMGB1), which acts on toll-like receptor 4 (TLR4), is required for the presentation of antigens from dying tumor cells. In addition, the autophagy-dependent release of ATP from dying cells causes the purinergic P2Y2-dependent chemotaxis of cells from the myeloid lineage and the P2RX7-dependent activation of the NLRP3 inflammasome in DC [1]. Kroemer postulated that the immune system determines the long-term success of anti-cancer therapies, and that this immune response is dictated by immunogenic tumor cell death. Indeed, he demonstrated results from the immunohistochemical characterization of human breast cancer specimens suggesting that signs of immunogenic cell death do have a major prognostic impact.

Viral vectors for cancer therapy represent a promising approach to treat tumors because of their ability for both killing cancer cells and awakening the immune system to attack the tumor. Cristian Smerdou (Center for Applied Medical Research (CIMA), Spain) presented extensive work with a Semliki Forest virus (SFV) replicon expressing interleukin-12 (IL-12; SFV-IL-12). In subcutaneous transplanted tumor models in mice, SFV-IL-12 had shown a curing efficacy greater than 90%. In contrast, its efficacy was reduced in spontaneous hepatocellular carcinoma (HCC) models. Smerdou showed that it is possible to increase the SFV-IL-12 effector response by combining the vector with an agonist mAb for CD137. Anti-CD137 synergized powerfully with the viral vector, promoting a great expansion of functional CD8+ T cells, while reducing dramatically SFV neutralizing antibodies, opening a door to multiple reinjections of the vector [2].

IFNα is a pleiotropic cytokine that plays an important role in the generation of immune responses. Sandra Hervás-Stubbs (Center for Applied Medical Research (CIMA), Spain) focused her talk in two critical topics: the contribution of IFNα for naïve CD8+ T cell priming and the importance of host type I IFN response induced by viral vectors carrying therapeutic transgenes used in experimental cancer treatment. Hervás-Stubbs showed how the presence of IFNα during CD8+ T cell priming increased responsiveness to homeostatic cytokines and recall antigens*,* providing a method to more efficiently generate human cytotoxic T lymphocytes (CTLs) from the naïve T-cell repertoire [3,4]. Intratumor replication of SFV genomes generates large quantities of ssRNA and dsRNA intermediate forms that can be sensed by host cells, leading to IFN-I expression. When IFN-α signaling was blocked (either genetically or by using neutralizing antibody), SFV-IL-12 anti-tumor efficacy mediated by CTLs was completely lost. These results emphasize the fact that type I IFN signaling could be crucial for the clinical benefit with viral vectors delivering therapeutic genes and cytopathic virotherapy (manuscript in preparation).

David Escors from Navarrabiomed, in Spain, presented vaccine strategies using DCs as therapeutic agents. Silencing of PD-L1 in DCs during lymphocyte priming induced a hyperactivation of T cells, mounting a faster response against tumors, but without any improvement of the final outcome of mice. Next, Escors constructed a panel of lentivectors expressing a cytokine gene, a shRNA against PD-L1 and a tumor antigen. A lentivector expressing IL-12 and TRP-1 as tumor antigen offered the best results following intratumoral delivery. Escors concluded that it was more important to inhibit PD-L1 expression and express IL12 both in immune and non-immune cells (e.g. in tumor cells). A novel method of culture/differentiation of myeloid derived suppressor cells (MDSCs) was presented and MDSCs transduced with the lentivector codifying for IL-12 plus PD-L1 shRNA and TRP-1 became efficient antigen presenting cells (APCs), leading to *in vitro* IFNγ expression by CD8+ T cells (manuscript in preparation).

David Sancho (Centro Nacional de Investigaciones Cardiovasculares Carlos III (CNIC), Spain) showed data on the role of the C-type lectin DNGR-1 (Clec9a) in the generation of immunity against viral infections. DNGR-1 is expressed selectively in the main subset of DC which mediates crosspriming. DNGR-1 expression on DCs and sensing of F-actin in dead cell debris was shown to be crucial for the generation of immunity against dead-cell associated antigens. Sancho`s recent work in a vaccinia virus model that showed the crucial contribution of DNGR-1 is at the level of cross-presentation of dead-cell associated antigen. Current work is focusing on the analysis of the secondary response to vaccinia vaccines, which is also impaired in the absence of DNGR-1. These results suggest that tissue damage sensed via DNGR-1 contributes to the efficacy of vaccines by means facilitating antigen cross-presentation [5].

### Experimental tumor imaging in immunotherapy

Two talks of the symposium were focused on the most novel in vivo imaging techniques to give light to the mechanisms of immunotherapy at cellular and tissue level. In this regard, Bettina Weigelin (Radboud University Nijmegen, The Netherlands) presented a model to image CTLs in vitro and in vivo. Time-lapse recordings obtained by dynamic imaging of 3D collagen matrices containing tumor cells and antigen-specific CTL allowed to dissect the different phases of CTL-mediated killing. The approach helped to identify a CTL crowd-based, serial killing mechanism dependent on sequential CTL-tumor cell interactions and the accumulation of sub-lethal hits to overcome melanoma cell resistance to CTL mediated apoptosis. The cooperation between multiple CTL acted as a compensation mechanism to maintain CTL killing efficiency if individual CTL contacts were weakened by factors of the tumor microenvironment. Intravital multiphoton microscopy (IVM), a powerful method for the exploration of biological processes in the physiological tissue context, enables monitoring cell migration and tumor dynamics. Spectacular movies with this technique showed that tissue interfaces guided tumor cell invasion and promoted the formation of collective, finger-like tumor cell strands infiltrating the healthy tissue. The same pro-invasive tissue tracks enhanced the frequency of OT1 CTL interactions with B16 melanoma cells causing preferential targeting of invading tumor cells by CTL. Furthermore, imaging of adoptive OT-1 CTL therapy and CD137 co-stimulation with administered mAb demonstrated that the combined therapy approach prolonged the cytotoxic phase of CTL resulting in a more focused and efficacious CTL effector phenotype in vivo (manuscript in preparation).

Philippe Bousso (Institut Pasteur, France) showed data of in vivo imaging of NK cytotoxic mechanisms in solid tumors and the effect of anti-tumor antibody immunotherapy. Dynamic In Situ Cytometry (DISC) combines IVM with flow cytometry-like multiparametric phenotypic analyses. Bousso demonstrated that NK cells interacted more transiently with their targets than CTL and NK tumor infiltration was less constrained by tumor cell density. The distinct dynamics of CTL and NK cells during killing reflected difference in calcium signaling upon interaction with their target. The studies of NK cell–mediated lysis indicated that NKG2D engagement promotes degranulation whereas FcR- signals play a critical role in NK-to-target contact stability [6]. Bousso also presented results of the characterization of in vivo mechanisms of anti-CD20 antibody therapy in B cell malignancies obtained IVM techniques. Bousso’s group studies indicate that the liver plays a central role in anti-CD20-mediated B cell depletion, and such depletion is contingent on B cell circulation. Anti-CD20 therapy induces circulating B cells to arrest in liver. Effectiveness of treatment is critically dependent on Kupffer cells (KC) [7]. Moreover, intravital imaging of Csfr1gfp/+Cd19cre/+Rosa26RFP/+Eμ-myc+/− mice developing spontaneous lymphomas reveals engulfment of tumor B cells by KC in liver sinusoids upon anti-CD20 mAb injection.

### Immunostimulatory monoclonal antibodies

CD137 (4-1BB) is a costimulatory receptor expressed on various immune system cells upon activation, including T and NK cells, and also on tumor endothelial cells. CD137 activation renders multiple effects such as: apoptosis resistance, proliferation, and gains in effector functions, antigen presentation and cytokine production. Little is known about its intimate mechanism of signal transduction from this. Ignacio Melero (Center for Applied Medical Research (CIMA), Spain) showed how CD137 activation leads to a TRAF-2 recruitment to the receptor and the formation of K63 polyubiquitin chains attached to TRAF2 and other substrates. K63 ubiquitin-ligase activity elicited CD137 crosslinking was crucial for downstream activation of NF-κB. Interestingly CD137 is internalized to endosomes following crosslinking but keeps its signaling function from such CD137 endosomes. Importantly, signaling is regulated by the deubiquitinases CYLD and A20. Of note, TRAF2 dominant-negative transgenic mice exhibited a delay in tumor rejection when treated with anti-CD137 mAb (manuscript in preparation). Melero’s talk continued with immunotherapy in a mouse model of spontaneous c-myc/OVA hepatocellular carcinoma (HCC). A triple mAb combination (anti-CD137 + anti-PD-L1 + anti-OX40, called Combo3) extended survival of the mice and this was even more efficacious when further combined to adoptive T cell therapy [8]. Melero also presented results about a new triple fusion protein constituted for Sushi domain of IL-15Rα, IL-15 and Apolipoprotein A-I. This chimeric protein had an extended half-life and a superior antimetastatic efficacy compared to other forms of IL-15 in tumor-bearing mice [9].

Bruno Paiva (Center for Applied Medical Research (CIMA), Spain) reviewed the spectrum of monoclonal gammopathies, including the malignant stage of multiple myeloma (MM). Despite significant therapeutic efforts MM remain an incurable disease and therefore several drugs with new mechanisms of action, including cellular immunotherapeutic strategies, are being developed to improve anti-myeloma immunity. MAb-based therapies function by ADCC or CDC by NK or T cells. On this subject, elotuzumab mediates ADCC in preclinical models. Importantly, ADCC activity of elotuzumab against MM cells is enhanced by lenalidomide, providing the framework for an ongoing phase III combination trial. Integration of novel agents into the transplant and non-transplant settings has significantly prolonged patients’ survival; persistent minimal residual disease (MRD) is often observed and translates into significantly inferior PFS and OS. In this regard, Paiva presented results suggesting that this small chemoresistant clones (MRD cells) might show unique genomic and phenotypic characteristics, including higher PD-L1 expression. Conversely, MM patients with long-term disease control have a constellation of unique immune changes favoring both immune cytotoxicity and recovery of B-cell production and homing, suggesting improved immune surveillance. In summary, these data will hopefully drive rational development of immunotherapeutic strategies for MM patients.

Immunotherapy for cancer treatment has won powerful allies in the recent years with the development of immunostimulatory mAbs. Especially relevant is the case of ipilimumab, an anti-CTL-associated antigen 4 (CTLA-4) antibody that blocks the inhibitory activities of this molecule expressed by activated T-lymphocytes and regulatory T cells. This therapeutic agent is the first ever exhibiting a survival improvement in metastatic melanoma. However, the finding of a predictive biomarker to identify patients who might benefit from ipilimumab treatment remains as a major unmet goal, as was claimed by Paolo Ascierto (National Tumor Institute Fondazione G. Pascale, Italy). The search for biomarkers has focused mainly in blood and tumor tissues [10]. The search for biomarkers has focused mainly in blood and tumor tissues. Among other candidates, absolute lymphocyte count (ALC), tumoral immuno infiltrate evaluation (TIL, FoxP3, IDO), upregulation of inducible costimulator (ICOS) molecule in T cells or an elevation of circulating Ki67^+^EOMES^+^CD8^+^ T cells stand out. All of them correlate with clinical benefit and overall survival (OS) after ipilimumab administration in retrospective studies. Besides, infiltration of M1 or M2 macrophages can influence the outcome of this treatment. CD124 and CD206 are markers for M2 phenotype, and their presence correlates with disease recurrence in preclinical model. Ascierto also made comments about new possible biomarkers in the form of genetic polymorphisms in CTLA-4 gene. It has been seen that responders to ipilimumab treatment share certain polymorphisms and non-responders do not. This preliminary finding represents a new exciting way to predict success with ipilimumab treatment that needs to be explored more deeply.

The study of the effects exerted by immunomodulatory mAbs has often been focused on T cells, but their molecular targets can usually be also found on other leukocyte subsets such as NK cells. These lymphocytes can mediate their cytotoxic effects on target cells directly or by means of antibody-dependent cell-mediated cytotoxicity (ADCC). This kind of induced cell death is the basis of the therapeutic effects of anticancer antibodies rituximab (anti-CD20, used against lymphomas), trastuzumab (anti-HER2, breast cancer) and cetuximab (anti-EGFR, head and neck cancers). Holbrook Kohrt, from Stanford University (USA), presented interesting work from bench to bedside, starting from the observation that the three antibodies enumerated above induced the expression of CD137 on NK cells, only in the presence of the corresponding antigen-positive tumor. Kohrt and Levy moved first to in vitro experiments and checked that the addition of anti-CD137 augmented ADCC mediated by NK cells. These results were further confirmed in nude mice bearing tumors from human origin [11,12]. Results in patients show a heterogeneous induction of CD137 on NK cells after treatment with antitumor mAbs. Kohrt identified five factors contributing to heterogeneity: tumor histology, time post-antitumor mAb therapy, tumor burden, span of prior antitumor mAb treatment and FcγRIIIa allelic polymorphism. Regarding the last factor, those alleles with less affinity for IgG Fc induced weaker CD137 expression on the NK cells.

Holbrook ended his talk showing promising preliminary data of several new rituximab combinations, including a phase I/II clinical trial combining rituximab with urelumab (anti-CD137), a phase I/II clinical trial in combination with a humanized anti-PD-1 mAb, and preclinical results in combination with anti-CD47 (“don’t eat me” signal). The results obtained are very exciting and begin a new era in immunotherapy targeting the innate immune response.

### Novel therapeutic agents for cancer immunotherapy

Tumors can counteract immune system surveillance by a variety of mechanisms, including the subversive use of cytokines and the exploitation of immunosupressor abilities of cells such as Regulatory T cells (Tregs) and myeloid-derived suppressor cells. Noelia Casares (Center for Applied Medical Research (CIMA), Spain) proposed strategies to inhibit Foxp3, a master regulator of Tregs, through the use of cell-penetrating peptide inhibitors. Two peptides were obtained from different approaches: the first of them (P60) using a phage-displayed random peptide library, the other one (Foxp3-393) based on the crystal structure of NFAT1:Foxp3:DNA complex. P60 inhibited murine and human-derived Treg activity and in vivo administration improved antitumor and antiviral immunotherapies [13]. Foxp3-393 was shown to function as a decoy peptide, disrupting NFAT:Foxp3 interaction, resulting in Treg activity reduction. On the contrary, this peptide resulted in functional CD40L expression on T cells and enhanced production of cytokines such as IL-2, IFN-γ or IL-17 in response to TCR stimulation (manuscript in preparation).

Tumors exploit the lymphatic vasculature for regional metastasis. Several cytokines and signaling receptors are involved in lymphangiogenesis, like vascular endothelial growth factor receptor 3 (VEGFR-3). Marisol Soengas (Spanish National Cancer Research Centre (CNIO), Spain) presented a VEGFR-3-GFP-Luciferase-reporter transgenic mouse model for in vivo imaging of tumor-associated lymphangiogenesis. This lymphatic vessel reporter mouse was shown very useful to monitor new lymphatic vessels associated to grafted or spontaneous tumors. For instance, tumors induce VEGFR-3 in draining lymph nodes thereby preconditioning a niche for eventual metastasis. Soengas’ group has identified BO-110 as a new pro-autophagy and apoptosis inducer in melanoma cells. BO-110 consists of synthetic long dsRNA nanocomplexes that can be sensed by TLR3 and MDA-5, inducing autophagy and caspase activation in melanoma cells, but not in normal melanocytes [14,15]. BO-110 has been tested both *in vitro* and *in vivo*, showing a potent antitumoral effect in different tumor models, inhibiting metastasis and lymphatic vessels formation too. In addition, BO-110 exhibits several effects in immune system cells including transition from M2 (protumorigenic) to M1 (antitumorigenic) macrophages. BO-110 also induced PD-L1 expression in melanoma cells, opening the door to combinations with monoclonal antibodies against PD-1 or PD-L1.

Oligonucleotide-based aptamer ligands represent a promising alternative to monoclonal antibodies (mAb) as tools to enhance immune responses and for counteracting immunosuppression. Fernando Pastor (Center for Applied Medical Research (CIMA), Spain) presented three effective approaches with aptamers in immunotherapy [16]. The first one was based on interference with non-sense mediated mRNA decay (NMD). siRNAs interfering with this cellular system were conjugated to an oligonucleotide aptamer that binds to prostate-specific membrane antigen (PSMA) for tumor targeting. Knocking down NMD in tumor cells resulted in mRNAs containing premature termination codons and aberrant proteins leading to tumor antigenicity. Next, Pastor presented an anti-CD28 aptamer engineered to function as an agonist as a strategy to potentiate anticancer immune responses. Administration in combination with anti-idiotype vaccine increased survival in subcutaneous A20 lymphoma-bearing mice. A third approach presented was a 4-1BB aptamer ligand conjugated to a tumor-targeting aptamer. Systemic administration of this chimeric aptamer resulted in a tumor-specific immune response against subcutaneous CT26 mouse models in a 4-1BB and PSMA-dependent manner.

Luis Álvarez-Vallina (Hospital Universitario Puerta de Hierro, Spain) updated new antibody platforms that are emerging as very promising scaffolds for immunotherapy and anti-angiogenesis. For these purposes the trimerbody platform was presented [17,18]. Trimerbodies are composed of single-chain variable fragments (scFv) connected though flexible linkers to the N- and /or C-terminus of a collagen-derived trimerization subdomain. Álvarez-Vallina disclosed the results of anti-human carcinoembryonic antigen (CEA) scFv-based N-trimerbody for tumor targeting and imaging. Furthermore, Álvarez-Vallina exposed the effects of a VEGF–blocking human single-domain antibody-mimetic (VB-SDAM)-based N-terminal trimerbody on VEGF-mediated endothelial cell proliferation and migration in vitro. Trimerbodies offer multiple possibilities to generate multivalent and/or multispecific molecules precisely engineered to meet specific target and requirements.

## Conclusion

The sessions were attended by about 130 participants. At least 64 of them were aged under 30. Questions and conversations indicated an excellent level among young professionals of immune-oncology including graduate students and residents. The future is theirs and they will deliver the promise of cancer immunotherapy as it is turning into successful reality. A talented young female resident told one of the authors during a coffee break: “This stuff seems to work, but only sometimes. It looks quite difficult to predict in spite of the beauty of the mechanisms. The field is surely full of unknowns and unknown unknowns” (translated from the original Spanish). She got it right, we think that this is a great scientific conclusion, to summarize the presentations.

Pamplona (Spain) provided an attractive environment for the meeting in which moderate doses of excellent wines, good humor and the participation of young investigators forecast the birth and consolidation of productive collaborations which are already fostering translational research efforts in cancer immunotherapy.

## Competing interests

IM is a consultant for BMS, MerckSerono, Genentech, Miltenyi Biotec and Dignabiotech. MAA and JIQ declare that they have no competing interests.

## Authors’ contributions

MAA, IM, and JIQ wrote and revised the manuscript. All the authors read and approved the final manuscript.

## References

[B1] Kroemer G Galluzzi L Kepp O Zitvogel L Immunogenic cell death in cancer therapy Annu Rev Immunol 2013 31 51 72 10.1146/annurev-immunol-032712-100008 23157435

[B2] Quetglas JI Dubrot J Bezunartea J Sanmamed MF Hervas-Stubbs S Smerdou C Melero I Immunotherapeutic synergy between anti-CD137 mAb and intratumoral administration of a cytopathic Semliki Forest virus encoding IL-12 Mol Ther 2012 20 9 1664 1675 10.1038/mt.2012.56 22735380 PMC3437571

[B3] Hervas-Stubbs S Riezu-Boj JI Gonzalez I Mancheño U Dubrot J Azpilicueta A Gabari I Palazon A Aranguren A Ruiz J Prieto J Larrea E Melero I Effects of IFN-alpha as a signal-3 cytokine on human naive and antigen-experienced CD8(+) T cells Eur J Immunol 2010 40 12 3389 3402 10.1002/eji.201040664 21108462

[B4] Hervas-Stubbs S Riezu-Boj JI Mancheño U Rueda P Lopez L Alignani D Rodríguez-García E Thieblemont N Leclerc C Conventional but Not Plasmacytoid Dendritic Cells Foster the Systemic Virus-Induced Type I IFN Response Needed for Efficient CD8 T Cell Priming J Immunol 2014 [Epub ahead of print] 10.4049/jimmunol.1301440 PMC4105236 24973449

[B5] Iborra S Izquierdo HM Martínez-López M Blanco-Menéndez N Reis e Sousa C Sancho D The DC receptor DNGR-1 mediates cross-priming of CTLs during vaccinia virus infection in mice J Clin Invest 2012 122 5 1628 1643 10.1172/JCI60660 22505455 PMC3336985

[B6] Deguine J Breart B Lemaître F Bousso P Cutting edge: tumor-targeting antibodies enhance NKG2D-mediated NK cell cytotoxicity by stabilizing NK cell-tumor cell interactions J Immunol 2012 189 12 5493 5497 10.4049/jimmunol.1202065 23183896

[B7] Montalvao F Garcia Z Celli S Breart B Deguine J Van Rooijen N Bousso P The mechanism of anti-CD20-mediated B cell depletion revealed by intravital imaging J Clin Invest 2013 123 12 5098 5103 10.1172/JCI70972 24177426 PMC3859399

[B8] Morales-Kastresana A Sanmamed MF Rodriguez I Palazon A Martinez-Forero I Labiano S Hervas-Stubbs S Sangro B Ochoa C Rouzaut A Azpilikueta A Bolaños E Jure-Kunkel M Gütgemann I Melero I Combined immunostimulatory monoclonal antibodies extend survival in an aggressive transgenic hepatocellular carcinoma mouse model Clin Cancer Res 2013 19 22 6151 6162 10.1158/1078-0432.CCR-13-1189 24030703

[B9] Ochoa MC Fioravanti J Rodriguez I Hervas-Stubbs S Azpilikueta A Mazzolini G Gúrpide A Prieto J Pardo J Berraondo P Melero I Antitumor immunotherapeutic and toxic properties of an HDL-conjugated chimeric IL-15 fusion protein Cancer Res 2013 73 1 139 149 10.1158/0008-5472.CAN-12-2660 23149919

[B10] Ascierto PA Kalos M Schaer DA Callahan MK Wolchok JD Biomarkers for immunostimulatory monoclonal antibodies in combination strategies for melanoma and other tumor types Clin Cancer Res 2013 19 5 1009 1020 10.1158/1078-0432.CCR-12-2982 23460532

[B11] Kohrt HE Houot R Goldstein MJ Weiskopf K Alizadeh AA Brody J Müller A Pachynski R Czerwinski D Coutre S Chao MP Chen L Tedder TF Levy R CD137 stimulation enhances the antilymphoma activity of anti-CD20 antibodies Blood 2011 117 8 2423 2432 10.1182/blood-2010-08-301945 21193697 PMC3062409

[B12] Kohrt HE Colevas AD Houot R Weiskopf K Goldstein MJ Lund P Mueller A Sagiv-Barfi I Marabelle A Lira R Troutner E Richards L Rajapaska A Hebb J Chester C Waller E Ostashko A Weng WK Chen L Czerwinski D Fu YX Sunwoo J Levy R Targeting CD137 enhances the efficacy of cetuximab J Clin Invest 2014 124 6 2668 2682 10.1172/JCI73014 24837434 PMC4089447

[B13] Casares N Rudilla F Arribillaga L Llopiz D Riezu-Boj JI Lozano T López-Sagaseta J Guembe L Sarobe P Prieto J Borrás-Cuesta F Lasarte JJ A peptide inhibitor of FOXP3 impairs regulatory T cell activity and improves vaccine efficacy in mice J Immunol 2010 185 9 5150 5159 10.4049/jimmunol.1001114 20870946

[B14] Tormo D Checińska A Alonso-Curbelo D Pérez-Guijarro E Cañón E Riveiro-Falkenbach E Calvo TG Larribere L Megías D Mulero F Piris MA Dash R Barral PM Rodríguez-Peralto JL Ortiz-Romero P Tüting T Fisher PB Soengas MS Targeted activation of innate immunity for therapeutic induction of autophagy and apoptosis in melanoma cells Cancer Cell 2009 16 2 103 114 10.1016/j.ccr.2009.07.004 19647221 PMC2851205

[B15] Alonso-Curbelo D Soengas MS Self-killing of melanoma cells by cytosolic delivery of dsRNA: wiring innate immunity for a coordinated mobilization of endosomes, autophagosomes and the apoptotic machinery in tumor cells Autophagy 2010 6 1 148 150 10.4161/auto.6.1.10464 19923903

[B16] Gilboa E McNamara J 2nd Pastor F Use of oligonucleotide aptamer ligands to modulate the function of immune receptors Clin Cancer Res 2013 19 5 1054 1062 10.1158/1078-0432.CCR-12-2067 23460536

[B17] Cuesta AM Sánchez-Martín D Sanz L Bonet J Compte M Kremer L Blanco FJ Oliva B Alvarez-Vallina L In vivo tumor targeting and imaging with engineered trivalent antibody fragments containing collagen-derived sequences PLoS One 2009 4 4 e5381 10.1371/journal.pone.0005381 19401768 PMC2670539

[B18] Blanco-Toribio A Sainz-Pastor N Alvarez-Cienfuegos A Merino N Cuesta AM Sánchez-Martín D Bonet J Santos-Valle P Sanz L Oliva B Blanco FJ Alvarez-Vallina L Generation and characterization of monospecific and bispecific hexavalent trimerbodies MAbs 2013 5 1 70 79 10.4161/mabs.22698 23221741 PMC3564888

